# Cell Wall Polysaccharide-Mediated Cadmium Tolerance Between Two *Arabidopsis thaliana* Ecotypes

**DOI:** 10.3389/fpls.2020.00473

**Published:** 2020-05-13

**Authors:** Yan Xiao, Xiuwen Wu, Dong Liu, Junyue Yao, Guihong Liang, Haixing Song, Abdelbagi M. Ismail, Jin-Song Luo, Zhenhua Zhang

**Affiliations:** ^1^Southern Regional Collaborative Innovation Centre for Grain and Oil Crops in China, College of Resources and Environmental Sciences, Hunan Agricultural University, Changsha, China; ^2^National Centre of Oilseed Crops Improvement, Hunan Branch, Changsha, China; ^3^International Rice Research Institute, Metro Manila, Philippines

**Keywords:** cadmium tolerance, *Arabidopsis thaliana* ecotypes, phytoremediation, cell wall polysaccharides, pectin, cellulose, hemicellulose

## Abstract

Cadmium (Cd) is a toxic metal element and the mechanism(s) underlying Cd tolerance in plants are still unclear. Increasingly more studies have been conducted on Cd binding to plant cell walls (CW) but most of them have focused on Cd fixation by CW pectin, and few studies have examined Cd binding to cellulose and hemicellulose. Here we found that Cd binding to CW pectin, cellulose, and hemicellulose was significantly higher in Tor-1, a Cd tolerant *A. thaliana* ecotype, than in Ph2-23, a sensitive ecotype, as were the concentrations of pectin, cellulose, and hemicellulose. Transcriptome analysis revealed that the genes regulating CW pectin, cellulose, and hemicellulose polysaccharide concentrations in Tor-1 differed significantly from those in Ph2-23. The expressions of most genes such as pectin methyl esterase inhibitors (*PMEIs*), pectin lyases, xyloglucan endotransglucosylase/hydrolase, expansins (*EXPAs*), and cellulose hydrolase were higher in Ph2-23, while the expressions of cellulose synthase-like glycosyltransferase 3 (*CSLG3*) and pectin ethyl esterase 4 (*PAE4*) were higher in Tor-1. The candidate genes identified here seem to regulate CW Cd fixation by polysaccharides. In conclusion, an increase in pectin demethylation activity, the higher concentration of cellulose and hemicellulose, regulated by related genes, in Tor-1 than in Ph2-23 are likely involved in enhanced Cd CW retention and reduce Cd toxicity.

## Introduction

Cadmium (Cd) is a toxic, heavy metal element that hinders plant growth and causes potential health risks to humans through the food chain (Hu et al., 2013; Åkesson et al., 2014). It is listed among the top 10 most dangerous substances by the Agency for Toxic Substances and Disease Registry (Benton et al., 2011). When excess Cd enters the cytoplasm of plant cells, it causes toxicity by triggering many oxidative stress reactions, such as lipid peroxidation, protein denaturation (Rellán-Álvarez et al., 2006), and increased reactive-oxygen species (Jia et al., 2016), which affect plant growth. Therefore, it is important to thoroughly understand the tolerance mechanisms of Cd in plants.

Cadmium accumulation in plant shoots mainly depends on Cd uptake by roots, sequestration within root vacuoles, and transportation and redistribution from roots to shoots (Ismael et al., 2019). Plant tolerance to Cd is based on various mechanisms, such as Cd chelation by cell wall (CW) components, vacuole compartmentalization, and Cd chelation by cytoplasmic organic acids or peptides (Memon and Schröder, 2009; Zhang J. et al., 2018; Deng et al., 2019).

The CW is a major storage site for heavy metals (Lux et al., 2013). It acts as the first barrier against Cd stress, as well as a protective barrier of protoplasts, mainly by directly trapping Cd outside the cell, which reduces cellular damage due to Cd toxicity (Richter et al., 2017). The primary CW is mainly composed of cellulose, hemicellulose, and pectin; some functional proteins, and a small amount of aromatic compounds (Showalter, 1993; Chebli and Geitmann, 2017). Among them, pectin polysaccharides modified by pectin methyl esterases (*PMEs*) and pectin acetyl esterases (*PAEs*) represent the most important CW components capable of binding to Cd (Peng et al., 2017), and pectin methyl esterase inhibitors (*PMEIs*) were reported to inhibit PMEs activity (Sénéchal et al., 2015). However, other polysaccharides in CWs also play important roles in metal binding and accumulation, such as cellulose and hemicellulose (Nishizono et al., 1987; Wan and Zhang, 2012). Data from previous studies showed that pectin polysaccharide levels in plant CWs increased under Cd stress (Meyer et al., 2015). Exogenous nitric oxide can enhance Cd tolerance in rice plants by increasing the pectin and hemicellulose contents in their root CWs (Xiong et al., 2009). In monocotyledonous and dicotyledonous non-grass plants, hemicellulose is mainly composed of xyloglucan. The xyloglucan endotransglucosylase/hydrolase enzyme (encoded by the *XTH* gene) catalyzes the hydrolysis and transglycosylation of xyloglucan polymers in plant CWs (Maldonado-Mendoza et al., 2005). Previous findings have shown that the *XTH* gene family may play important roles in alleviating metal toxicity in plants (Zhu et al., 2012). Loss of *XTH31* function resulted in enhanced aluminum tolerance (Zhu et al., 2014) and the *xth15* transfer-DNA mutant was more resistant to aluminum than the wild type (Zhu et al., 2013a). Although little research has described cellulose and Cd binding, a previous study showed that the Cd content in plant cellulose increased significantly after Cd treatment (Wang P. et al., 2018).

Recent studies on the CW retention of Cd have mainly focused on pectin, and few studies have been conducted to investigate hemicellulose and cellulose binding to Cd (Parrotta et al., 2015). In the present study, we found that genes such as *PAEs*, pectin lyases, *XTHs*, and cellulose synthase-like glycosyltransferase 3 (*CSLG3*) families participate in the regulation of pectin modification, as well as cellulose and hemicellulose polysaccharide levels. Based on transcriptome analysis, we also found these candidate genes may participate in regulating Cd chelation to the CW by altering the Cd concentration in different cellular components. As so, Cd could accumulate more in plant CWs, thereby reducing Cd toxicity to cellular organelles and providing a theoretical basis for Cd phytoremediation.

## Materials and Methods

### Plant Materials

*Arabidopsis thaliana* ecotypes Ph2-23 and Tor-1 were provided by Dr. Chao Daiying, Institute of Plant Physiology and Ecology, Shanghai Academy of Life Sciences, Chinese Academy of Sciences.

### Growth Conditions

The two *Arabidopsis thaliana* ecotypes were seeded in a vegetative soil culture pot in a greenhouse (300 mmol photons m^-2^s^-2^, 16-h photoperiod, 22°C), sealed with a membrane to retain water, and the seeds germinated 3 days later. When the *A. thaliana* plants had grown two true leaves, the seedlings were transferred to a 4-L pot for hydroponic culture. The nutrient solution contained 1.25 mM KNO_3_, 0.625 mM KH_2_PO_4_, 0.5 mM MgSO_4_, 0.5 mM Ca(NO_3_)_2_⋅4H_2_O, 0.025 mM Fe-EDTA, 0.25 ml L^–1^ micronutrients (70 mM H_3_BO_3_, 14 mM MnCl_2_, 1 mM ZnSO_4_, 0.5 mM CuSO_4_, and 0.2 mM NaMoO_4_). The nutrient solution was renewed every 4 days. After 20 days of hydroponic growth, plants in the experimental group were treated with 10 μM CdCl_2_ for 4 days, and those in the control group were not treated with Cd.

### Determination of Chlorophyll, Proline, and Malondialdehyde (MDA) Concentrations

Twenty-day-old *A. thaliana* plants were treated with or without 10 μM CdCl_2_ for 4 days and their rosette leaves (approximately 0.5 g) were extracted with 80% acetone and left in the dark for 24 h (Wang T. et al., 2018). The extracts were used for measuring absorption at 645 and 663 nm with a spectrophotometer. Chlorophyll *a*, chlorophyll *b*, and total chlorophyll concentrations were calculated as described by Arnon (1949). Proline concentrations were measured using ninhydrin colorimetry as described by Bates et al. (1973). Briefly, shoot tissues (0.5 g) were sampled, ground in 5 mL of 3% sulfosalicylic acid, and centrifuged at 22,000 × *g* for 5 min. The supernatant of each sample was collected, and proline concentration was determined after reaction with acid indene. The MDA concentration was determined by the thiobarbituric acid method (Esterbauer et al., 1991; Wang T. et al., 2018). Briefly, after full grinding in 5% trichloroacetic acid, the supernatant was centrifuged for 10 min at 3000 × *g*. Then, 2 mL of the supernatant was thoroughly mixed with 2 mL 0.67% thiobarbituric acid. The sample was then bathed at 100°C for 30 min. The absorbance of each supernatant was measured at 450, 532, and 600 nm after centrifugation.

### Determination of Dry Weight, Total Cd Content, and Metal Concentrations Under Low Temperature

Twenty-day-old *A. thaliana* plants were treated with or without 10 μM CdCl_2_ for 4 days, after which their roots and shoots were harvested and washed with 0.1 mM CaCl_2_ for 1 min, followed by four times rinsing with deionized water, as described by Jian et al. (2019), and then dried in an oven to a constant mass (dry weight). For the total Cd assay, 20-day-old *A. thaliana* plants were transferred to Cd-free or 10 μM CdCl_2_ solutions for 4 days. After this period, the whole plants were collected, rinsed with deionized water four times and dried to constant weight. Then, 1 mL nitric acid was added, and both samples were digested in a boiling water bath for 2 h. Each sample was diluted 100-fold with deionized water (Jian et al., 2019), and the total Cd concentration and other metal concentrations were determined by inductively coupled plasma mass spectrometry (ICP-MS) on a NexION^TM^ 350X instrument (PerkinElmer, Massachusetts, United States). Total Cd content was calculated as total Cd concentration × dry mass; primary transport index (PTI) was calculated following Moral et al. (1994) as shoot_*metal concentrations*_/root_*metal concentrations*_.

After 20 days of normal culture, ecotypes Ph2-23 and Tor-1 were incubated with 10 μM CdCl_2_ for 30 min in a 4°C refrigerator or at room temperature (22°C), as reported previously (Luo et al., 2018). Their roots were then sampled and dried to constant weight. The Cd concentration was determined by ICP-MS after digestion with nitric acid as described above.

### Extraction of Subcellular Components and Determination of Cd Concentrations

Twenty-day-old *A. thaliana* plants were treated with or without 10 μM CdCl_2_ for 4 days. The subcellular components were extracted by differential centrifugation (Weigel and Jäger, 1980). Briefly, fresh samples (0.5 g) were mixed with 8 mL extracting agent (250 mmol/L sucrose, 50 mmol/L Tris-HCl pH 7.5, 1 mmol/L dithiothreitol) and ground on ice to form homogenates. Each homogenate was centrifuged at 300 × *g* for 30 s to remove the residual CW fraction. Then, the samples were filtered and centrifuged at 20,000 × *g* for 45 min to precipitate the organelles. The extracted CWs and organelles were dried in an oven, digested in a 1:4 mixture (vol/vol) of HNO_3_ and HClO_4_, and diluted 100-fold with deionized water. The soluble fraction was again diluted 10-fold with deionized water, and Cd concentration was determined by ICP-MS.

Cell wall fractions were extracted as described by Brummell et al. (2004). The samples were quickly homogenized in 80% ethanol and incubated for 20 min at 90°C in a water bath. After cooling, the samples were centrifuged at 6000 × *g* for 10 min, and the precipitates were washed once with 1.5 mL 80% ethanol and 1.5 mL acetone (swirling for 2 min and centrifugation for 10 min at 6000 × *g*). Then, the supernatant was discarded, and the precipitate was soaked for 15 h in 1 mL dimethyl sulfoxide (for starch removal) and centrifuged for 10 min at 6000 × *g*. The supernatant was then discarded, and the CW precipitate dried for further use.

### Determination of Cd in Pectin, Cellulose, and Hemicellulose

Pectin, cellulose, and hemicellulose were extracted according to Wu et al. (2017). The CW fraction was obtained from each sample and mixed with 5 mL 50 mM sodium acetate buffer containing 50 mM trans-1,2-diaminocyclohexane-*N,N,N′,N′*-tetraacetic acid (CDTA) at pH 6.5. After shaking at 24°C for 12 h, the samples were centrifuged at 10,000 × *g* for 20 min. The residue was washed with 3 mL deionized water before centrifugation (5,000 × *g*, 10 min), and the supernatant comprised CDTA-soluble pectin. The residue was mixed with 5 mL 50 mM sodium carbonate solution, shaken for 12 h, and centrifuged at 10,000 × *g* for 20 min. The residue was washed with 3 mL water and centrifuged at 5,000 × *g* for 10 min. The supernatant comprised Na_2_CO_3_-soluble pectin. The residue was shaken with 5 mL 4 M KOH (containing 1% NaBH_4_) for 3 h and centrifuged at 5,000 × *g* for 20 min. The residue was washed with 3 mL water before centrifugation (5,000 × *g*, 10 min). The supernatant was the hemicellulose fraction, and the precipitate the cellulose fraction. After the cellulose precipitate was dried to a constant weight, 3 mL nitric acid was added for digestion. In parallel, 1 mL solution of CDTA-soluble pectin, Na_2_CO_3_-soluble pectin and hemicellulose, which was extracted previously, was digested with 3 mL nitric acid. The resulting solution was diluted 100-fold with deionized water. Metal concentrations were determined by ICP-MS with a NexION^TM^ 350X instrument (PerkinElmer).

### Determination of Pectin Methylesterase (PME) Activity and Pectin, Cellulose, and Hemicellulose Concentrations

Pectin concentrations were detected using the Pectin Assay Kit (Shanghai Zcibio, Co., Ltd., China). Initially, each shoot sample (0.1 g) was homogenized by grinding with 5 mL distilled water, followed by low temperature centrifugation at 8000 × *g* for 10 min. Reagents I and II were incubated at 37°C for 10 min before beginning the assays. One hundred microliters of each sample was mixed with 100 μL reagent II. The control tubes contained 100 μL of each sample and 100 μL distilled water. The standard tube was prepared by mixing 100 μL reagent II with 100 μL reagent I, whereas the control tube was prepared by mixing 100 μL reagent II with 100 μL distilled water. The mixture of each tube was diluted to 800 μL with concentrated sulfuric acid, and the absorption at 530 nm was read after incubation at 95°C for 5 min in a water bath.

A Pectinesterase Assay Kit (Suzhou Comin Biotechnology, Co., Ltd., China) was used to detect PME activity as follows. Shoots (1 g/sample) were fully ground in 2 mL extraction reagent and centrifuged at 12,000 × *g* and 4°C for 15 min, after which each supernatant was transferred to a 15-mL centrifuge tube. Then, 50 μL reagent II and 8 mL reagent I were added, mixing after each addition, and then the pH was adjusted to 7.8 with reagent IV. Each centrifuge tube was placed in an oven at 37°C for 60 min, the pH was adjusted to 7.8 every 20 min, and the amount of reagent IV consumed was recorded. One unit of enzymatic activity (U) was defined as 1 μmol of NaOH consumed per minute by each tissue.

Cellulose concentrations were detected using a Cellulose Assay Kit (Beijing Solarbio Science & Technology Co., Ltd., China) and the extracted CW samples (approximately 5 mg each) as described by Brummell et al. (2004). The cellulose was homogenized with 0.5 mL distilled water, and the volume was adjusted to 0.5 mL. Then, each sample was placed in an ice-water bath, and 0.75 mL of concentrated sulfuric acid was added slowly, incubated for 30 min, and centrifuged at 8000 × *g* for 10 min. Then, 300 μL supernatant was mixed with 70 μL of a mixture of reagent I and reagent II, 630 μL concentrated sulfuric acid was added, and the resulting solution was incubated in a water bath at 95°C for 10 min, cooled to room temperature, and the absorbance measured at 620 nm.

Hemicellulose concentrations were detected using the Hemicellulose Assay Kit (Beijing Solarbio Science & Technology Co., Ltd.). Initially, 20-day-old *A. thaliana* plants were treated with or without 10 μM CdCl_2_ for 4 days. The shoots were dried to a constant weight, ground thoroughly, and passed through a 40-mesh sieve. After adding 1 mL 80% ethanol and vortexing, each sample was incubated in a water bath at 90°C for 10 min and centrifuged at 8,000 × *g* for 10 min. After washing each precipitate with distilled water, samples were dried to a constant weight. Then, 0.5 mL reagent I and 0.5 mL reagent II were added sequentially, and the samples were incubated in a water bath at 90°C for 1 h, mixed well, and centrifuged at 8,000 × *g* for 10 min. To each supernatant sample (125 μL) 125 μL reagent II and 750 μL distilled water were added, and the samples were incubated at 90°C for 5 min and cooled to room temperature before measuring their absorbance at 540 nm.

### RNA Extraction and Determination of Gene Expression Levels

Cadmium-treated roots and shoot samples (0.2 g) were placed in liquid nitrogen, and total RNA was extracted with TRIzol (Ambion, Inc., Austin, United States). Complementary DNA (cDNA) templates were then synthesized using the HiScript II 1st Strand cDNA Synthesis Kit (Vazyme Biotech Co., Ltd., Nanjing, China). Relative gene expression levels were determined by quantitative real-time PCR (qRT-PCR) using SYBR Premix Ex-Taq^TM^ II (Takara Bio Inc., Kusatsu, Japan). The sequences of the primers used in the assays are shown in Supplementary Table S1, and the specific primers for the qRT-PCR analysis were designed using Primer Premier 6.0 software^1^.

### Transcriptome Sample Preparation and Analysis

Shoots and roots of Ph2-23 and Tor-1 plants grown for 20 days and sampled after Cd treatment for 3 days were used for transcriptome analysis. We employed the mixed-sampling method (Kawahara et al., 2012), with each biological replicate included three samples of the same treatment. Biological replicates were quickly submerged in liquid nitrogen and sent to Gene *Denovo* Biological Sequencing Company (Guangzhou, China) for RNA-sequencing (RNA-Seq) analysis. We use R^2^ to carry out the Principal Component Analysis (PCA). Go function annotation of the identified proteins was carried out in GO database^3^. This process can be summarized as sequence alignment, mapping, calculation, hypergeometric tests, and annotation augmentation. The data are presented in a heat map, which was prepared using GraphPad Prism 8^4^. The Venn diagram was generated using a publicly available website^5^.

### Statistical Analyses

We performed minimum differential multiple-range comparisons to analyze our data, with each experiment being carried out with at least four biological replicates. *P* < 0.05 was considered to reflect a significant difference, and *P* < 0.01 was considered to reflect a highly significant difference. Analyses were conducted in SPSS software^6^. All bar charts were prepared with GraphPad Prism 8.

## Results

### Ecotype Tor-1 Coped Better With Cd Stress Than Ecotype Ph2-23

**FIGURE 1:**
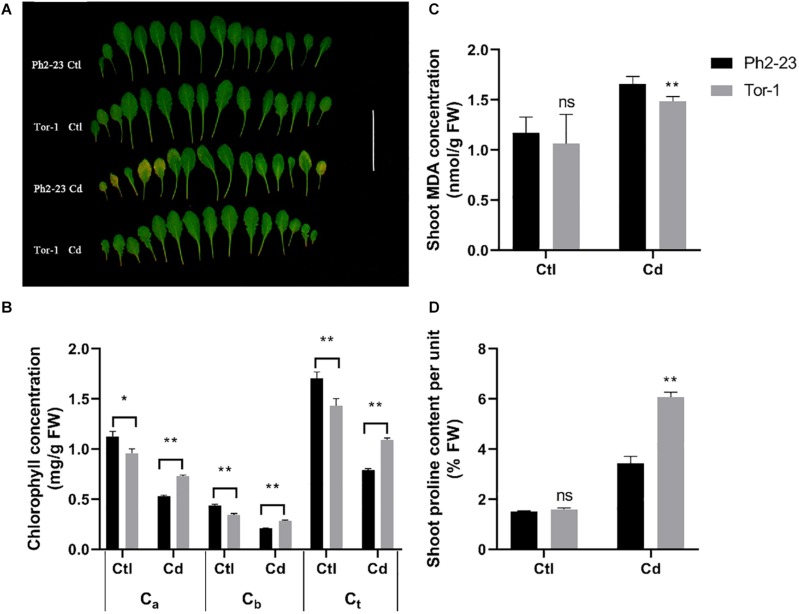
Differences in Cd tolerance of two *Arabidopsis* ecotypes treated with or without 10 μM CdCl_2_ for 4 days. **(A)** Photograph of leaves showing that Ph2-23 was more sensitive to Cd stress than Tor-1. **(B)** Chlorophyll loss under Cd^2+^ compared to control. C_*a*_: Chlorophyll *a*; C_*b*_: Chlorophyll b; C_*t*_: total Chlorophyll. **(C)** Shoot malondialdehyde (MDA) concentration in the two ecotypes under control and Cd treatment. **(D)** Percentage of shoot proline content under control and Cd treatment. Ctl means control treatment, with normal culture conditions; Cd means treatment with CdCl_2_. Data are means of four measurements (*n* = 4), and the vertical bars indicate the SD. One (*) and two (**) asterisks indicate significant differences from the control at *P* < 0.05, and 0.01, respectively, ns, differences are not significant.

After 4 days of Cd treatment, the leaves of *A. thaliana* ecotype Ph2-23 became progressively more yellowish during Cd treatment, while no difference was found between the two ecotypes under control (without) Cd treatment (Figure 1A). Compared with those in Tor-1, the concentrations of chlorophyll *a* (C_*a*_), chlorophyll *b* (C_*b*_), and total chlorophyll (C_*t*_) in Ph2-23 significantly decreased following exposure to Cd toxicity (Figure 1B). Under control conditions, the MDA concentration in the shoots of Ph2-23 and Tor-1 plants were similar, but after Cd treatment, the MDA concentration in Ph2-23 was significantly higher than that in Tor-1 (Figure 1C). After Cd treatment, the proline concentration in Tor-1 was significantly higher than that in Ph2-23, whereas no differences were observed between the two ecotypes under the control treatment (Figure 1D). Based on the observed phenotype and physiological indicators measurements, Tor-1 seems to cope better with the constraints of Cd exposure than Ph2-23.

### Ecotype Ph2-23 Translocated Less Cd to the Shoots Than Tor-1

**FIGURE 2:**
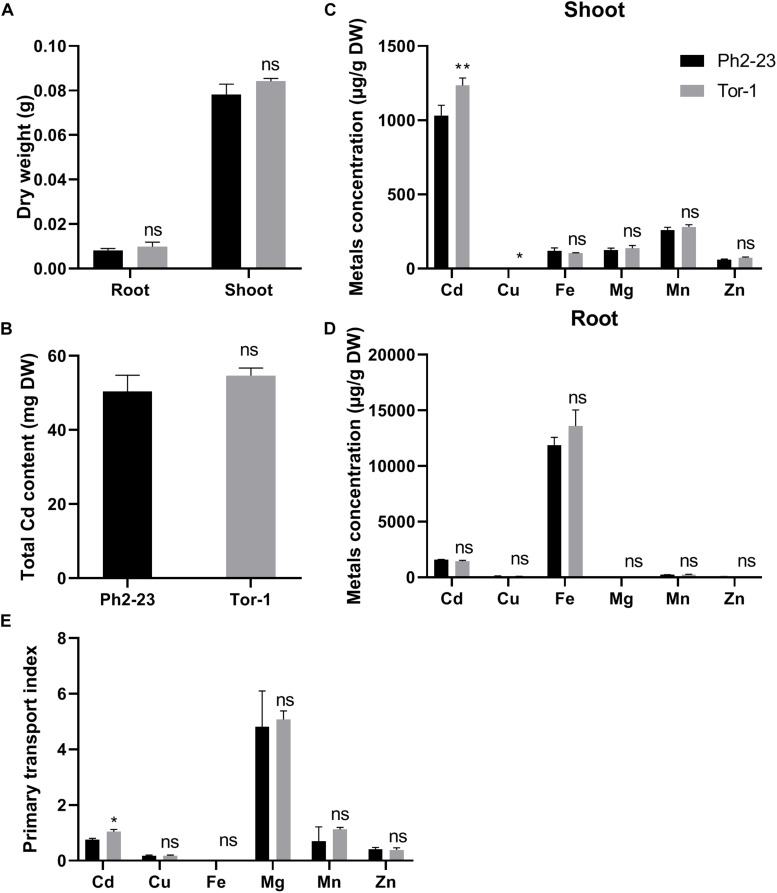
The uptake and distribution of Cd in two *Arabidopsis* ecotypes. **(A)** Effects on dry weight under 10 μM CdCl_2_ treatment. **(B)** Total Cd content in two ecotypes with or without 10 μM CdCl_2_ treatment for 4 days. **(C)** Shoot and **(D)** Root mentals distribution. **(E)** Primary transport index of metals (shoot_*metal concentrations*_/root_*metal concentrations*_). Data are means of four measurements (*n* = 4), and the vertical bars indicate the SD. One (*) and two (**) asterisks indicate significant differences from the control at *P* < 0.05, and 0.01, respectively, ns, differences are not significant.

No significant difference was found in the dry weights of shoots and roots (Figure 2A) and total Cd concentration during Cd treatment between Ph2-23 and Tor-1 (Figure 2B). At both low (4°C) and normal (22°C) temperature treatments, the root absorption of Cd did not differ markedly between the two ecotypes after Cd treatment for 30 min (Supplementary Figure S1). The Cd concentration in the shoots of Tor-1 was significantly higher than that in the shoots of Ph2-23 (Figure 2C), but with no differences in roots (Figure 2D). In addition, no significant differences were observed in the concentrations of other metal elements (iron, magnesium, manganese, and zinc) between the shoots and roots of the two varieties, except for copper (Cu) concentration, which was lower in the shoots of Ph2-23 than that of Tor-1, while no differences were observed in root Cu concentrations. The primary transport index (PTI) of Cd in Tor-1 was higher than that in Ph2-23 but no differences were found between the two ecotypes in the PTI of other metals (Figure 2E). These data indicated that Tor-1 translocated more Cd to shoots, though was less affected by Cd stress.

### Cd Distribution in Cellular Fractions of Ph2-23 and Tor-1

**FIGURE 3:**
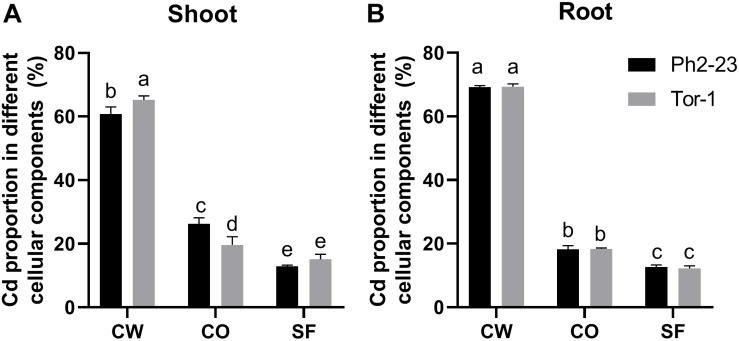
Distribution of Cd in the subcellular fractions of shoots **(A)** and roots **(B)** of the two Arabidopsis ecotypes grown under control or 10 μM CdCl_2_ treatment. CW, cell wall; CO, cellular organelles; SF, soluble fraction. Data are means of four measurements (*n* = 4), and different letters indicate means are significant differences (*P* < 0.05).

**FIGURE 4:**
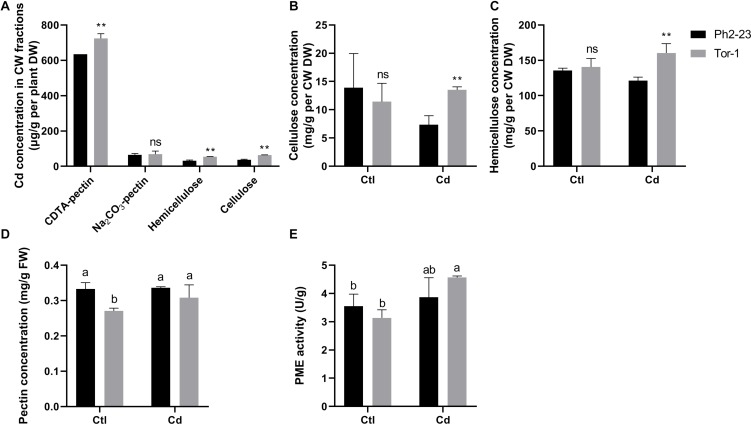
Cd concentration and changes of cell wall components between Ph2-23 and Tor-1 with or without 10 μM CdCl_2_ treatment for 4 days. **(A)** Cd concentrations of CW components. **(B–D)** Cellulose, hemicellulose, pectin concentration in shoots. **(E)** PME activity in shoots. Ctl means control treatment, with normal culture conditions; Cd means treatment with CdCl_2_. Data presented are means of four measurements (*n* = 4), and different letters indicate means are significantly different (*P* < 0.05), two (**) asterisks indicate significant differences from the control at *P* < 0.01, ns, differences are not significant.

In both ecotypes, most Cd accumulated in the CW, followed by organelles, and then by the soluble fraction (Figures 3A,B). More Cd was detected in the CWs of Tor-1 shoots and less Cd was present in the organelles (Figure 3A). The Cd concentrations in the different components of root cells did not differ significantly between the two ecotypes (Figure 3B). This suggests that the relatively high concentration of Cd in the shoot CW may be responsible for the difference observed in Cd tolerance of these two ecotype. Shoot Cd concentrations in the CW CDTA-pectin, hemicellulose, and cellulose in Tor-1 were significantly higher than those in Ph2-23, while no difference in Na_2_CO_3_-pectin associated Cd concentration observed between the two ecotypes (Figure 4A). No obvious differences were observed for the cellulose content between the two ecotypes under control conditions. However, after Cd treatment, the cellulose concentration in Tor-1 became significantly higher than that of Ph2-23 (Figure 4B), and similar trend was observed for the hemicellulose concentration (Figure 4C). Pectin concentration in Tor-1 increased after Cd treatment, but not in Ph2-23 (Figure 4D). Similar results were also found for PME activity (Figure 4E).

### Differentially Expressed Genes (DEGs) Between Ph2-23 and Tor-1

**FIGURE 5:**
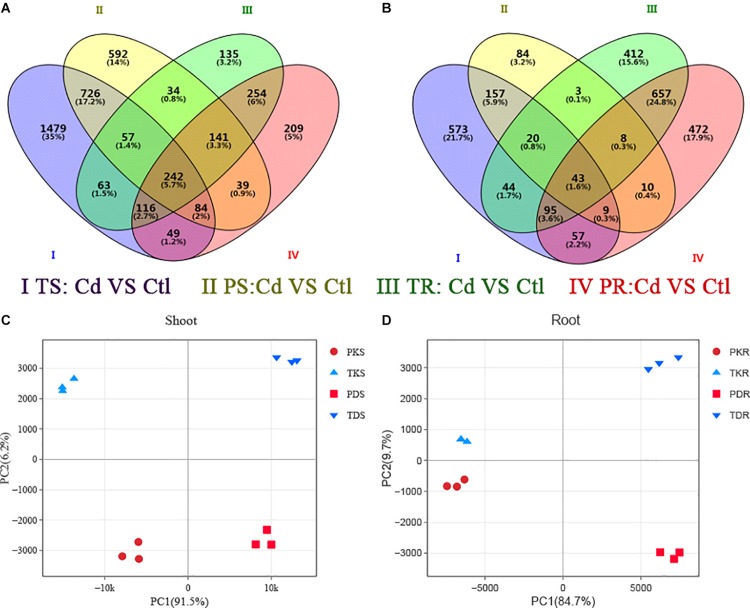
The transcriptome of differentially expressed genes (DEGs) in the two *Arabidopsis* ecotypes. Venn diagram analyses of **(A)** up-regulated and **(B)** down-regulated genes in two groups. P, Ph2-23; T, Tor-1; S, shoots; R, roots; K, control (Ctl); D, Cd. Data in each group are means of three replicates. Principal component analysis in shoot **(C)** and roots **(D)**, Ph2-23 and Tor-1 sample distributions according to PC1 and PC2 are shown, with three biological replications, the thresholds for selecting DEGs were | log_2_ FC| > 1, FDR < 0.05.

RNA-Seq analysis was performed to understand the molecular basis of the differential Cd sensitivity observed in the two *Arabidopsis* ecotypes. Compared to Ph2-23, Tor-1 showed more up-regulated genes (Figure 5A, Supplementary Figure S2, and Supplementary Table S2) and fewer down-regulated genes in shoots and roots following Cd exposure (Figure 5B, Supplementary Figure S2, and Supplementary Table S2). Both ecotypes shared 1109 up-regulated and 229 down-regulated Cd-responsive genes in the shoots (Figure 5A), and 753 up-regulated and 803 down-regulated Cd-responsive genes in the roots (Figure 5B). PCA showed that the first principal component (PC1) explained 95.1 and 84.7% while PC2 explained 6.2 and 9.7% of the variation in gene expression in the shoots (Figure 5C) and roots (Figure 5D) of the two cultivars, respectively.

**FIGURE 6:**
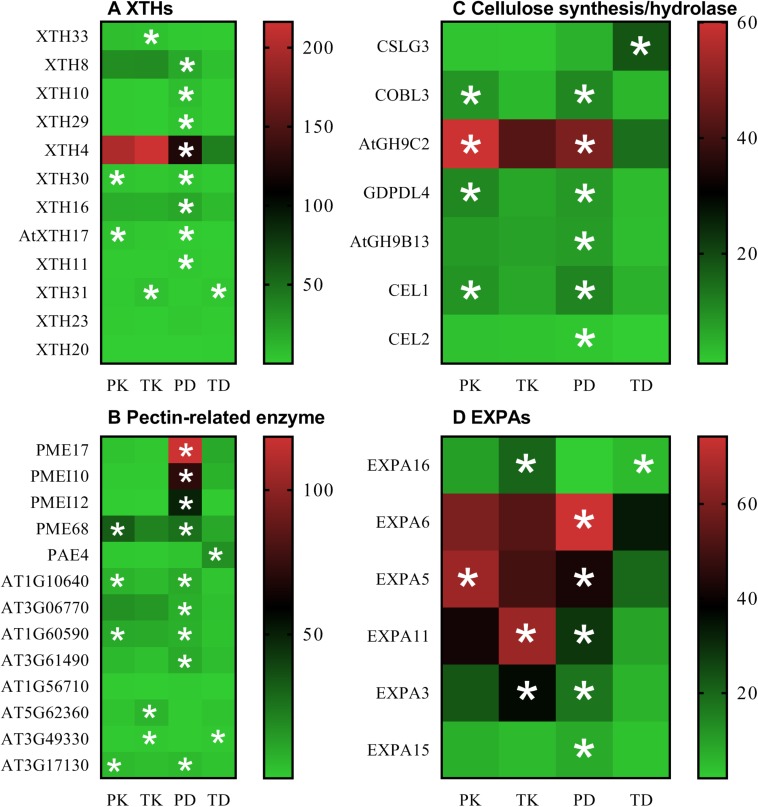
Transcriptome analysis of candidate DEGs under the control and Cd treatment in the two *Arabidopsis* ecotypes. **(A)** XTHs expression, **(B)** the gene expression of pectin-related enzyme, **(C)** the gene expression of cellulose synthesis/hydrolase, **(D)** EXPAs expression. PK and TK refer to Ph2-23 and Tor-1 under control treatment, respectively; PD and TD refer to Ph2-23 and Tor-1 following Cd treatment, respectively. Data presented means ± SE (*n* = 3). The thresholds for selecting DEGs were | log_2_ FC| > 1, FDR < 0.05, with one (*) asterisk indicates significant differences from the control at *P* < 0.05. Different colors indicate differential expressions, with red color indicating high expression, and green indicating low expression.

Gene Ontology (GO)-based enrichment analysis of the RNA-Seq data conducted on Tor-1 and Ph2-23 highlighted the DEGs representing enrichment of “CW” genes in the shoots (Supplementary Figure S3 and Supplementary Table S3). Taking into account the differences found in CW Cd accumulation and the GO enrichment analysis, we focused on the CW. We identified 60 DEGs (between Ph2-23 and Tor-1) involved in CW modification with a false-discovery rate (FDR) < 0.05. These DEGs encoded 31 proteins related to CW degradation, six proteins related to CW synthesis, and 22 other proteins involved in the CW (Supplementary Table S4). Genes related to CW degradation were more expressed in Ph2-23. In addition, Cd treatment induced 112 CW-related genes in Tor-1, compared with only 60 CW-related genes in Ph2-23 (Supplementary Table S4). Under Cd treatment, the expression of most *XTHs*, pectin lyases (*AT1G10640*, *AT3G06770*, *AT1G60590*, *AT3G61490*), PMEs (*PME17*, *PME68*), PMEIs (*PME17*, *PMEI10*, *PMEI12*), and one pectin methylesterase inhibitor superfamily protein (*AT3G17310*) were higher in Ph2-23 than in Tor-1 (Figures 6A,B and Supplementary Table S2); however, some pectin methylesterase inhibitor superfamily proteins (*AT5G62360*, *AT3G49330*) were significantly down-regulated after Cd treatment in Tor-1 (Figure 6B and Supplementary Table S2). The expression of *CSLG3* (involved in cellulose synthesis) was up-regulated after Cd treatment in Tor-1 compared to Ph2-23 and the expression of *COBL3* (also involved in cellulose synthesis) was higher in Ph2-23 with or without Cd treatment. Moreover, the expression levels of cellulose hydrolases (such as *ATGH9C2*, *ATGH9B13*, *CEL1*, and *CEL2*) were significantly lower in Tor-1 than in Ph2-23 (Figure 6C). We also found that the expression levels of *EXPAs* were higher in Ph2-23 than in Tor-1, except for *EXPA16*, which showed higher expression in Tor-1 under control treatment (Figure 6D).

**FIGURE 7:**
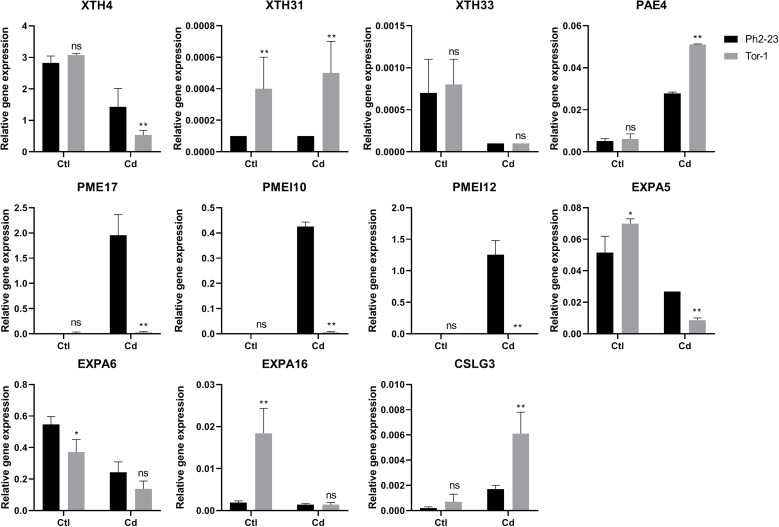
qRT-PCR expression data of genes in shoots and roots between Ph2-23 and Tor-1. Ctl means control treatment, with normal culture conditions; Cd means treatment with CdCl_2_. Data are means of four measurements (*n* = 4), and the vertical bars indicate the SD. One (*) and two (**) asterisks indicate significant differences from the control at *P* < 0.05, and *P* < 0.01, respectively, ns, differences are not significant.

We verified the accuracy of the transcriptomics data through qRT-PCR, and the correlations between the RNA-Seq data and qRT-PCR data was 0.9517 (Figure 7 and Supplementary Figures S4A,B, S5). The qRT-PCR data also confirmed that *XTH4*, *PAE4, PME17, PMEI12*, *PMEI10*, *EXPA5*, *EXPA6*, and *CALG3* were induced by Cd (Figure 7), consistent with our RNA-Seq data.

## Discussion

In this study, we found that *A. thaliana* ecotype Tor-1 is more tolerant to Cd toxicity than ecotype Ph2-23, based on their phenotypes and the chlorophyll concentrations (Figures 1A,B). MDA is the main secondary product generated by the peroxidation of polyunsaturated fatty acids and it can reflect the degree of damage to cellular membranes (Del Rio et al., 2005). Furthermore, higher plants can accumulate proline in response to external stresses (e.g., heavy metals, drought, or high salt), which can be beneficial in mitigating these stresses (Fabro et al., 2004; Haudecoeur et al., 2009). Less cellular toxicity was observed in Tor-1 than in Ph2-23 (Figures 1C,D), which led us to further investigate which mechanism(s) are likely underlying the difference in tolerance observed between these ecotypes. There was no difference in dry weight between Tor-1 and Ph2-23 but the Cd concentration in the shoots of Tor-1 was significantly higher than that of shoots of Ph2-23, suggesting more Cd had accumulated in the shoots of Tor-1 than in those of Ph2-23 (Figures 2A,C, 4A). However, no significant difference was observed in the total Cd content between the two ecotypes (Figure 2B), suggesting that the difference in tolerance might be related to genes associated with Cd transport or cellular compartmentation.

Plant tolerance to Cd may be regulated by various genes responsible for Cd uptake and transport. ATP-binding cassette (ABC)-type transporters can transport more Cd–PC complexes to vacuoles in *A. thaliana*, which reduces the toxicity of Cd in the cytoplasm (Brunetti et al., 2015). In addition, Cd can be transported directly to vacuoles via calcium/hydrogen inhibitors (*CAXs*), metal-transporter proteins (*MTPs*), and heavy metal ATPase 3 (*HMA3*), which are tonoplast-localized transporters (Menguer et al., 2013; Pittman and Hirschi, 2016; Shafiq et al., 2019). In the present study, the expression of *HMA2*, responsible for Cd/zinc transport, was higher in Tor-1 than in Ph2-23, and *CAX4* expression was higher in Tor-1 roots. Although more Cd might be transferred to vacuoles by *CAX4* in Tor-1 roots than in Ph2-23 roots, Cd translocation from the roots to the shoots of Tor-1 was also higher (Figure 2E), and the expression levels of *MTP1*, *HMA3*, *HMA4*, *ABCC1*, and *ABCC2* were not significantly different (Supplementary Figures S4A,B). These findings demonstrated that the differences in tolerance between Ph2-23 and Tor-1 were not primarily due to Cd-transport-related genes.

Previous reports showed that plants could chelate Cd through the CW during detoxification (Peng et al., 2017; Zhang et al., 2019). Cd can be fixed in CWs to reduce the toxicity of Cd to cellular organelles (Yu et al., 2019). Thus, we measured Cd concentrations in different cell fractions and found that the shoot CWs of Tor-1 contained more Cd, with less Cd allocated to organelles, and with no significant differences in roots (Figures 3A,B). Data from previous studies showed that exogenous auxin promoted *A. thaliana* hemicellulose synthesis, which reduces toxicity (Zhu et al., 2013b), and that the root CW polysaccharides of *Sedum alfredii* increased during Cd treatment, especially pectin and hemicellulose polysaccharides (Li et al., 2015). Further fractional extraction of the CW revealed that CDTA-pectin, cellulose, and hemicellulose fixed more Cd in Tor-1 (Figure 3C), suggesting that differences in their abundances in the CW may have caused the Cd concentration differences.

The degree of pectin acetylation can be regulated by *PAEs*, which may expose negatively charged groups, enabling them to bind Cd (Philippe et al., 2017). The demethyl esterification of *A. thaliana* pectin that can be modified by *PMEs*, *PMEI* was reported to inhibit PME activity (Röckel et al., 2008; Sénéchal et al., 2015), as was *PME17* (Sénéchal et al., 2014). Pectin lyases are a group of enzymes that are thought to contribute to the degradation of pectin (Cao, 2012). Among the DEGs in the CW after Cd treatment, *PAE4* expression was significantly higher in Tor-1; genes inhibiting PME activity were down-regulated in Tor-1, while genes inhibiting PME activity and pectin lyases were up-regulated in Ph2-23. The expressions of most *PMEIs*, as well as pectin lyases and PME inhibitor proteins, were significantly higher in Ph2-23 than in Tor-1. Furthermore, genes that inhibit PME activity were down-regulated in Tor-1 after Cd treatment, but not in Ph2-23 (Figure 6B). Increased pectin concentrations and PME activities were also found in Tor-1 compared with Ph2-23 (Figures 4D,E).

*XTHs* is known to be involved in hemicellulose synthesis in the primary CWs of higher plants and can play important roles in plant responses to metals such as aluminum and Cd (Zhu et al., 2013b; Han et al., 2014; Xuan et al., 2016). Our transcriptome analysis showed that *XTH*-expression levels were generally higher in Ph2-23 than in Tor-1 after Cd treatment, especially *XTH4* (Figures 6A, 7). In addition, after Cd treatment, the hemicellulose content of Ph2-23 was significantly lower than that of Tor-1 (Figure 3D). Thus, Tor-1 may sequester Cd in the CW by increasing the hemicellulose content through *XTHs* under Cd treatment. Hemicellulose was widely viewed as a potential substrate for the production of liquid fuels and other value-added materials (Sun et al., 2012), meaning that using these *XTH* candidate genes to increase the hemicellulose content in plants with large biomass might not only help plants tolerate Cd toxicity but might also be used for increasing their production.

*EXPAs* may work in conjunction with cellulase and pectin lyase to break down CWs (Medina-Escobar et al., 1997; Llop-Tous et al., 1999; Harrison et al., 2001), although the exact mechanism(s) whereby *EXPAs* act on Cd is unclear. Previous findings showed that *PtoEXPA12* over-expression (OX) increased Cd accumulation in tobacco plants (Zhang H. et al., 2018), although another study showed that *TaEXPA2* OX is involved in tobacco resistance to Cd (Ren et al., 2018). After *EXPA10* was knocked out, aluminum accumulation in the root hair region of rice plants decreased (Che et al., 2016). In the present study, *EXPAs* were significantly higher in Tor-1, except for *EXPA16* (Figures 6D, 7); therefore, we hypothesized that *EXPAs* might be involved in Cd tolerance, although further testing is required to confirm this hypothesis.

Plant cellulose is produced by cellulose synthases, such as cellulose synthase-like (CSL) enzymes, members of the *AtCesA* cellulose synthase family, and members of the COBRA-LIKE (COBL) gene family (Manfield et al., 2004; Ben-Tov et al., 2015); cellulose hydrolysis in plants is regulated by endo-1,4-beta-glucanase (Levy et al., 2002). Less cellulose was detected in Ph2-23 after Cd treatment than in Tor-1 (Figure 3D). Although the expression of *COBL2*, which is involved in cellulose synthesis, was also higher in Ph2-23 than in Tor-1, expression of the cellulose synthase gene *CSLG3* was much lower, and genes related to cellulose hydrolysis (such as *AtGH9C2*, *AtGH9B13*, and *CEL1*) were up-regulated (Figure 6C). These findings suggest that Tor-1 may regulate cellulose synthesis through these genes, leading to Cd binding. Cellulose is used in large quantities in fuel, and, like hemicellulose, whether these candidate genes can be transformed and/or overexpressed to enhance the synthesis of cellulose in plants with large biomass to achieve Cd tolerance and for cellulose production awaits further investigation.

## Conclusion

*Arabidopsis thaliana* ecotype Tor-1 accumulated more Cd in the shoots and was more tolerant of Cd than *A. thaliana* ecotype Ph2-23. The difference in tolerance between these two ecotypes mainly resulted from variation in polysaccharide concentrations in the CW. The main anti-Cd toxicity mechanism of Tor-1 may involve gene regulation (e.g., *XTH4*, *CSLG3, PME17*, *PAE4*) to increase the cellulose and hemicellulose contents of the CW and the degree of pectin modification (to increase the CW retention of Cd), thereby reducing Cd toxicity in cellular organelles. Our study provides a strong evidence for the importance of shoot CW polysaccharides in Cd tolerance and identified candidate genes that could potentially be used to enhance Cd tolerance.

## Data Availability Statement

The datasets generated for this study can be found in the NCBI using accession number PRJNA611457.

## Author Contributions

YX and ZZ designed the experiments and analyzed the data. YX performed most of the experiments. YX, J-SL, AI, and ZZ wrote the manuscript. All authors contributed to the initial design of the project, read and approved the manuscript.

## Conflict of Interest

The authors declare that the research was conducted in the absence of any commercial or financial relationships that could be construed as a potential conflict of interest.

## Footnotes

1 http://www.premierbiosoft.com/primerdesign/

2 http://www.r-project.org/

3 http://www.geneontology.org/

4 https://www.graphpad.com/scientific-software/prism/

5 https://bioinfogp.cnb.csic.es/tools/venny/index.html

6 https://www.ibm.com/analytics/spss-statistics-software
