# Supplementary material for: Cell Wall Polysaccharide-Mediated Cadmium Tolerance Between Two Arabidopsis thaliana Ecotypes
Source: Front Plant Sci. 2020 May 13;11:473. doi: 10.3389/fpls.2020.00473 (PMC7239314; doi:10.3389/fpls.2020.00473)
Supplement: Supplementary file 1 [file Table_1.pdf]

Table S1 Primers used for qRT-PCR analysis

**Primer name**	Sequence		Tissue
XTH4	XTH4-F	CAATGGTAATGGCTATTCCTC	S
	XTH4-R	CTGGTTGTCCTGTTCTGTT	
XTH31	XTH31-F	TCAAGTCTCTTCGTCCATAC	S
	XTH31-R	GGTCTCCAGGATGCTCTT	
XTH33	XTH33-F	AAGCCACGACGAGATAGA	S
	XTH33-R	ACGGAACAGCCACTTAAC	
EXPA6	EXPA6-F	TTCTACGGTGGCTCTGAT	S
	EXPA6-R	TTAGTCGCAGTGATGAAGAT	
EXPA5	EXPA5-F	GCTTCTCGTGGTTCATCT	S
	EXPA5-R	CCGTCTCCAGTCCATAAC	
EXPA16	EXPA16-F	GCCAAGGATACGGAACAA	S
	EXPA16-R	ACCAACGAGAACAGCATTA	
PDR8	PDR8-F	AGTGTCGCAGTACGGTGATG	S
	PDR8-R	CGCCACTGGTCCCATAAAGT	
ABCC1	ABCC1-F	TTTCTGTGCTTCGCTTCCCT	S
	ABCC1-R	GCTGTCCAGGTTCAATGGGA	
ABCC2	ABCC2-F	CTCGAGGGGAAACACGACAA	S
	ABCC2-R	CACTGCAAGCCCTTCTACCA	
MTP1	MTP1-F	GCACACTTGCCTTTTCGGTT	S
	MTP1-R	CCCTTTTCGAGCTTTGTGGC	
PAE4	PAE4-F	TATCCCCTCCGTTGACCGTA	S
	PAE4-R	CCGCCTCCACCCTCTAAATA	
CSLG3	CSLG3-F	AGAGTACCACGATGGAGCCT	S

	CSLG3-R	GTACGGATCAGCCGTGCATA	
PME17	PME17-F	TGAAAGCATGGTGCAGCCAAA	S
	PME17-R	GTCACGGCACTTTGGTCCTA	
PMEI10	PMEI10-F	CACCCTCTCATCCGCCAAAT	S
	PMEI10-R	TGAAGCGAAGTCACCGAGTC	
PMEI12	PMEI12-F	GTTTCGCAACCGCTCAAACT	S
	PMEI12-R	CGGTCTTCGTCGATGCTAGG	
CAX2	CAX2-F	TCCATGTTTGCGGTCCCATT	R
	CAX2-R	CAGTGATGAACAGCATCGCC	
CAX4	CAX4-F	ACCGCTTGTTTTGCCTTGTC	R
	CAX4-R	GGTTTCGGTTGAGGGTGAGT	
HMA2	HMA2-F	CGAGGTGCAAGTGCAACAAA	S/R
	HMA2-R	GTTCCAATGTCCAGGCCAGA	
HMA3	HMA3-F	GATGGCGGAAGGTGAAGAGT	R
	HMA3-R	GTGGACAACGATGACGGTTC	
HMA4	HMA4-F	TAAGCCAGTTCGCCGACTTT	R
	HMA4-R	TTCTCCTTGTCGTTTCGCCA	
Actin	Actin-F	GATGAGGATGATGCTTCTACG	S/R
	Actin-R	CCTGAGCGTTGTATCTTGGT	
