# Supplementary material for: Cell Wall Polysaccharide-Mediated Cadmium Tolerance Between Two Arabidopsis thaliana Ecotypes
Source: Front Plant Sci. 2020 May 13;11:473. doi: 10.3389/fpls.2020.00473 (PMC7239314; doi:10.3389/fpls.2020.00473)
Supplement: Supplementary file 5 [file Presentation_1.pdf]

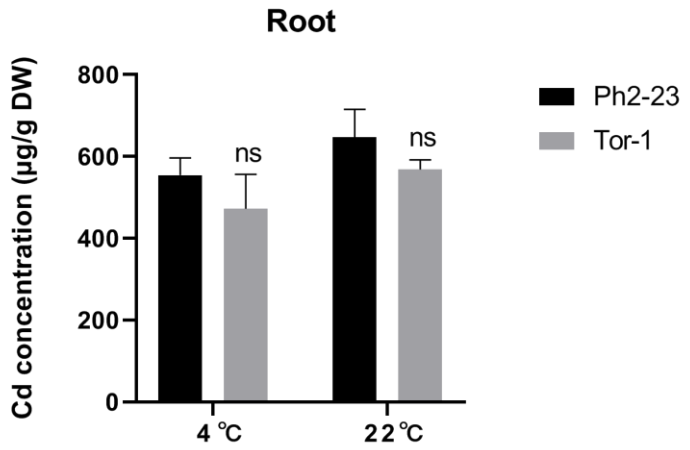

Supplementary Figure 1. Root Cd concentration in the two *Arabidopsis* ecotypes under 4ºC and 22ºC ,with 10 μM $CdCl_2$ treatment for 30 min. Data are means ± SE (n = 4), and vertical bars indicate the SD, ns = differences are not significant.

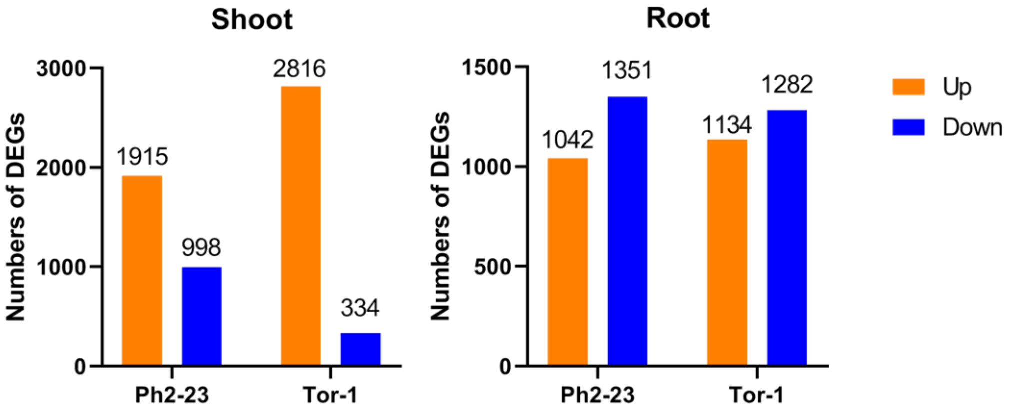

Supplementary Figure 2. Number of differentially expressed genes (DEGs) between Ph2-23 (cd vs ctl) and Tor-1 (cd vs ctl) in (A) shoot and (B) root, with three biological replications, the thresholds for selecting DEGs were $|\log_2 FC| > 1$, $FDR < 0.05$.

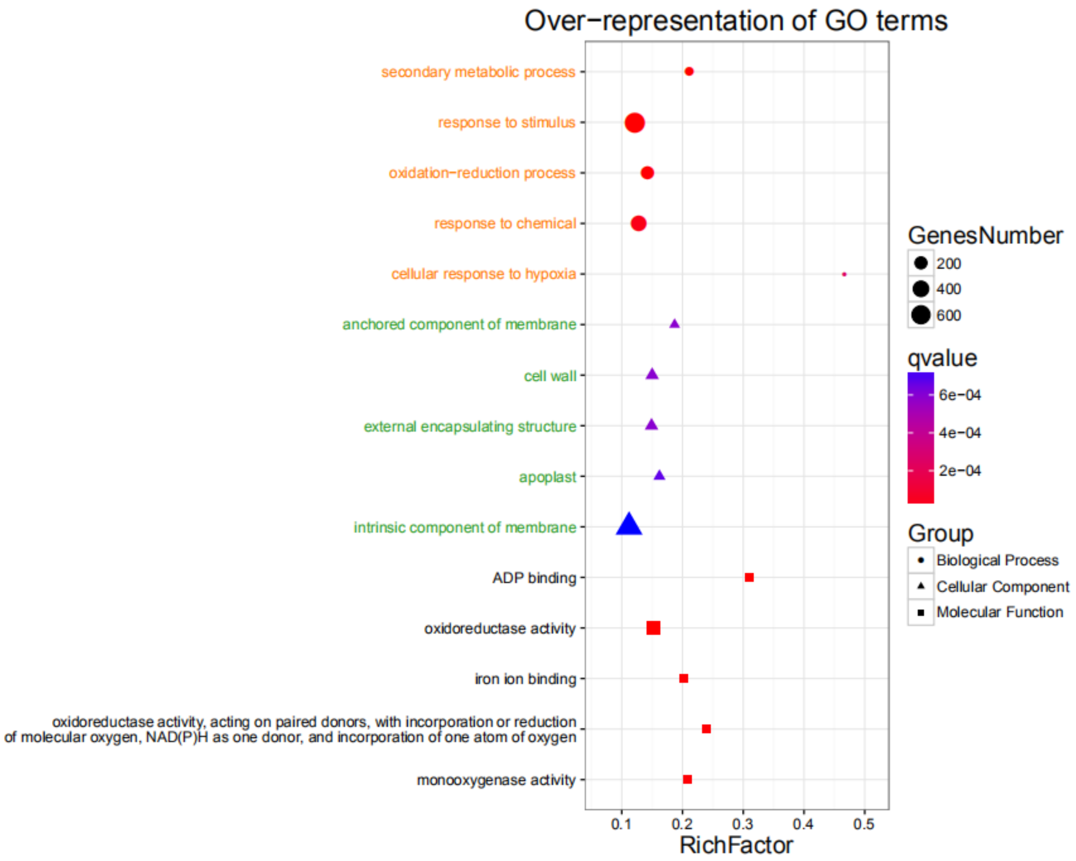

Supplementary Figure 3. Top 5 of go enrichment analysis (Tor-1-vs-Ph2-23). with three biological replications, the thresholds for selecting DEGs were $|\log_2 FC| > 1$, $FDR < 0.05$.

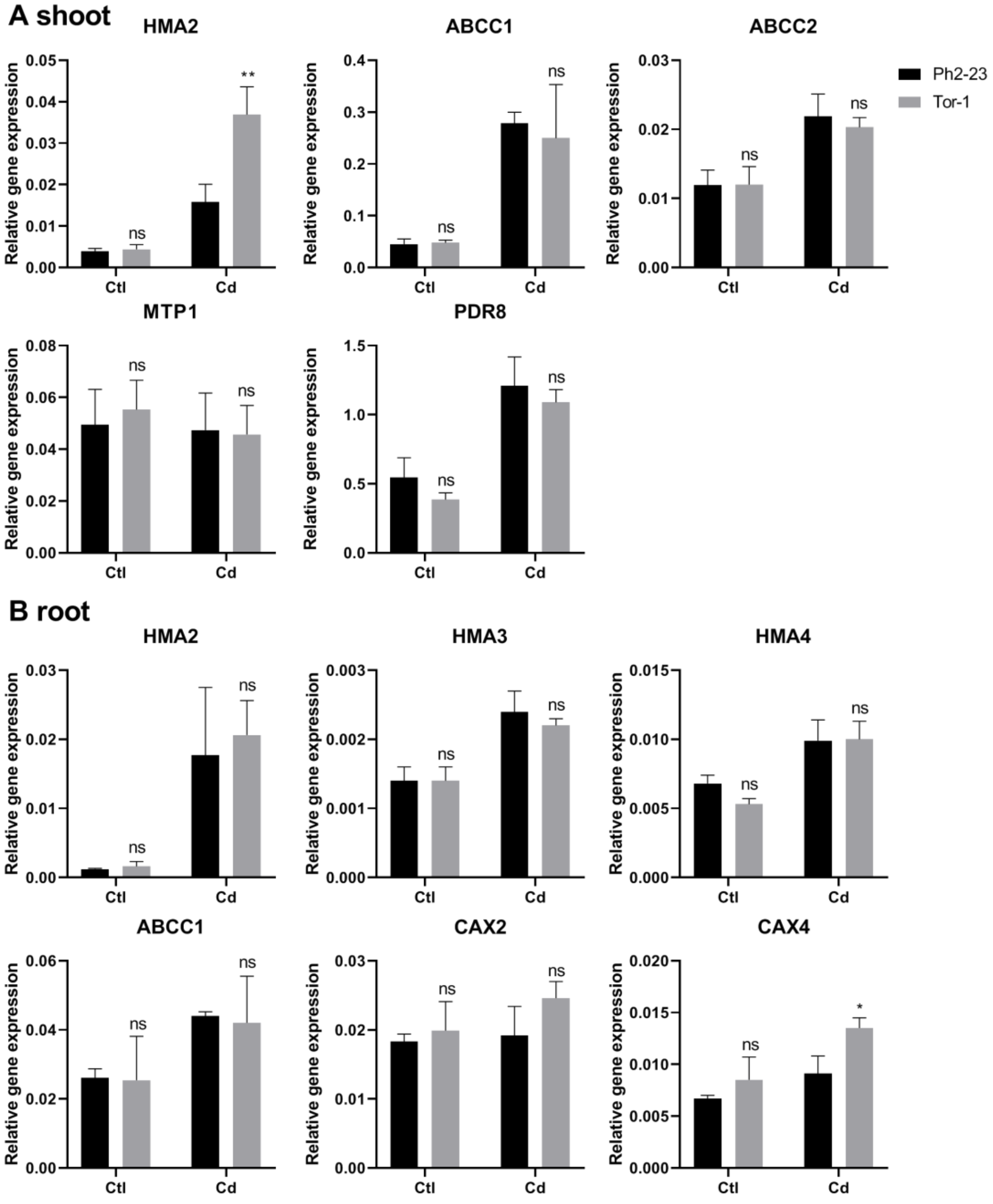

Supplementary Figure 4. qRT-PCR expression data of genes expression related to Cd transport in shoots (A) and roots (B) between Ph2-23 and Tor-1. Ctl means control treatment, with normal culture conditions; Cd, treatment with $CdCl_2$. Data are means ± SE (n = 4), and vertical bars indicate the SD. * and ** indicate significant differences from the control at $P < 0.05$ and 0.01, respectively; ns = differences are not significant.

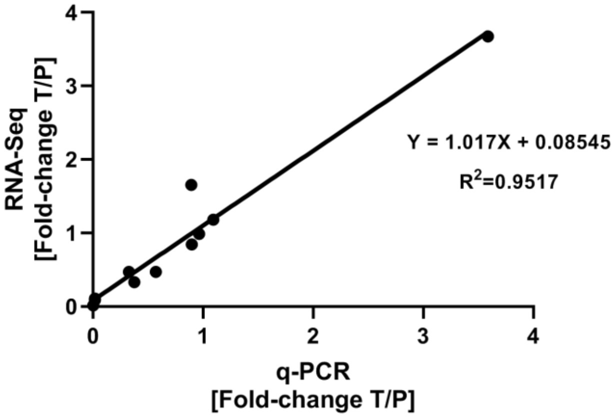

Supplementary Figure 5. Linear analysis between q-PCR and RNA-Seq results. T, Tor-1; P,Ph2-23.
